# A systems approach identifies co-signaling molecules of early growth response 1 transcription factor in immobilization stress

**DOI:** 10.1186/s12918-014-0100-8

**Published:** 2014-09-11

**Authors:** Nikolaos A Papanikolaou, Andrej Tillinger, Xiaoping Liu, Athanasios G Papavassiliou, Esther L Sabban

**Affiliations:** 1 Laboratory of Biological Chemistry, Department of Medicine, Aristotle University of Thessaloniki, Thessaloniki, 54124, Hellas (Greece); 2 Department of Biochemistry and Molecular Biology, New York Medical College, Valhalla 10595, NY, USA; 3 Current Address: Clyde and Helen Wu Center of Molecular Cardiology, Department of Physiology and Cellular Biophysics, Columbia University College of Physicians and Surgeons, New York 10032, NY, USA; 4 Department of Biological Chemistry, Medical School, University of Athens, 75 M. Asias Street, Athens, 11527, Hellas (Greece)

**Keywords:** Adrenal medulla, Egr1, Stat3, Prlh1, Networks, Stress

## Abstract

**Background:**

Adaptation to stress is critical for survival. The adrenal medulla, the major source of epinephrine, plays an important role in the development of the hyperadenergic state and increased risk for stress associated disorders, such as hypertension and myocardial infarction. The transcription factor Egr1 plays a central role in acute and repeated stress, however the complexity of the response suggests that other transcription factor pathways might be playing equally important roles during acute and repeated stress. Therefore, we sought to discover such factors by applying a systems approach.

**Results:**

Using microarrays and network analysis we show here for the first time that the transcription factor signal transducer and activator of transcription 3 (Stat3) gene is activated in acute stress whereas the prolactin releasing hormone (Prlh11) and chromogranin B (Chgb) genes are induced in repeated immobilization stress and that along with Egr1 may be critical mediators of the stress response.

**Conclusions:**

Our results suggest possible involvement of Stat3 and Prlh1/Chgb up-regulation in the transition from short to repeated stress activation.

## 1 Background

The adrenal medulla plays a key role in the response to acute and chronic stress. It is the major site of biosynthesis of epinephrine (Epi) in the periphery. Upon exposure to stress, the release of adrenomedullary Epi and norepinephrine (NE) are among the most rapid response to handle the emergency situation. This is crucial for activation of the “fight or flight” response to deal with a threat to homeostasis.

When stress is prolonged or repeated the adrenal medulla exhibits crucial adaptive and subsequently maladaptive responses. These include important changes in gene expression. The best characterized are the up-regulation of expression of catecholamine biosynthetic enzymes (reviewed in [1]). Exposure to single immobilization stress triggers a manifold elevation in transcription and expression of mRNAs of the catecholamine biosynthetic enzymes, tyrosine hydroxylase (TH), the first and major rate limiting enzyme in catecholamine biosynthesis as well as of phenylethanolamine N-methyltransferase (PNMT), the enzyme which catalyses the conversion of NE to Epi [2]–[4]. This rise is transient and returns to normal within one day and is not sufficient for substantial increase in their activity. Following repeated IMO stress the increase in gene expression of catecholamine biosynthetic enzymes is now more sustained with prolonged elevation in catecholamine biosynthetic enzyme activity (reviewed in [5]).

To determine the repertoire of changes and the mechanism of alterations in gene expression mediating the response of adrenal medulla to single and repeated stress, microarray profiling was performed [6]. Following single exposure to 2 hr IMO stress there was altered expression, of greater than 2 fold, in nearly 4% of the total transcripts. Transcription factors and cell signaling genes displayed the most prevalent changes. Approximately 20% of the transcripts up-regulated by single IMO were transcription factors. Not only was Egr*1* mRNA markedly induced in the adrenal medulla by single as well as repeated exposure to IMO, but pathway analysis indicated that *Egr1* likely plays a central role [6].

Egr1 (Zif268, NGFI-A, TIS8 or Krox24) is a transcription factor with three zinc fingers of the Cys2His2 class (reviewed by [7],[8]). Egr1 binds to a GC-rich motif (5′-GCG (T/G) GGGCG-3′) through its three zinc finger DNA binding domains [9] and modulates transcription of a number of genes that participate in various cellular functions (reviewed by [10],[11]). Egr1 plays critical roles in divergent cellular processes. For example, Egr1 and Stat3 have been implicated in neuronal differentiation, specifically during neurite outgrowth (reviewed in [12],[13], in tumor development [14]-[16], oxidant stress [17], immune responses [18] and in insulin signaling and in nutrition [19]).

Egr1 target genes include catecholamine biosynthetic enzymes. Transcription of both *TH* and *PNMT* is up-regulated by Egr1 [20]–[24]. We have previously shown that Egr1 is markedly induced in the adrenal medulla by IMO stress [25]. While barely expressed under basal conditions, immunofluorescence demonstrated widespread expression in the nucleus of TH expressing chromaffin cells in the adrenal medulla after IMO stress [26]. However the molecules that form the core of the signaling cascade inducing these responses are not well understood. Because complex biological behaviors arise from the coordinated behavior of sets of genes acting in concert (gene modules), we hypothesized that genes that are co-expressed with *Egr1* during single or repeated IMO stress might provide insights into to significant signaling pathways that participate in stress signaling. Here we employed Gene Set Enrichment Analysis to identify *Egr1* co-expressed genes from IMO microarrays, extracted their interactors and all their interrelationships and reconstructed Egr1 networks. From their network properties, we have identified the transcription factor Stat3 and the peptide Prlh1 in short and prolonged stress respectively as Egr1 neighbors in the adrenal medulla implicating them for the first time in stress signaling.

## 2 Results

### 2.1 Gene sets that enrich with Egr1 expression in acute and repeated stress

**Figure 1:**
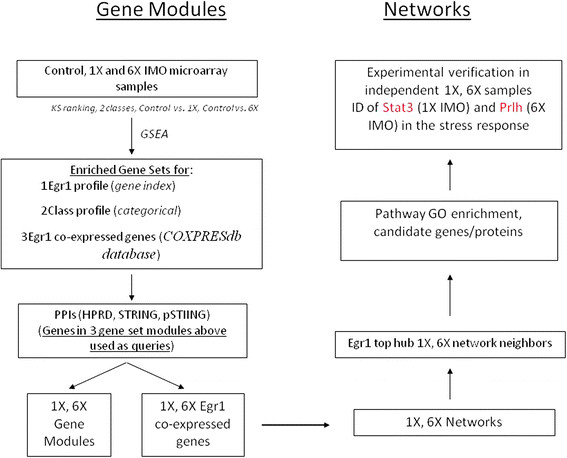
**Strategy for identifying gene sets, the top genes that enrich with** ***Egr1*** **up-regulation in rat adrenal medulla with single (1×) or repeated (6×) immobilization stress samples, network construction and use of network properties to identify novel pathways and genes active in stress.**

Αcute and repeated stress responses are accompanied by different patterns of gene expression, particularly of transcription factor genes, suggesting an interplay of transcription factors and the gene programs they control. In order to identify novel genes and their products that might be instrumental in networks leading from acute to repeated stress, we applied a strategy (Figure 1) that allowed us the re-construction of Egr1-centered networks and the extraction of network neighbors from expression profiles identical to that of Egr1. We executed this strategy in two steps: First, we used Kolmogorov-Smirnov analysis of acute (1×) and repeated (6×) IMO microarray expression data in order to rank expression levels of all genes in the microarrays. Second we used gene set enrichment analysis (GSEA) and computed gene module enrichment scores (ES) for each module of genes that are either co-expressed with Egr1 (positive ES) or anti-coexpressed (negative ES) (Additional file 1: Figure S1). Specifically, using Egr1 as an index gene in GSEA we extracted the top fifty genes (Egr1_POS module, top positive ES score, control vs. 1×) that are co-expression neighbors of *Egr1* (See Additional file 2, Computational and Bioinformatic Methods, for a full account of methods). Second, by categorical class analysis (control vs. 1×) we extracted the top fifty down-regulated genes and top fifty up-regulated genes in control and in 1× samples. We repeated the same analyses for 6× IMO data. Third, we extracted the top negatively correlated genes from the top gene sets (Egr1_NEG module, top negative ES scores). Finally, we mined a list of 300 rat genes from the COXPRESdb database reported to be co-expressed with Egr1 (Data are available online as Additional files 3 and 4).

### 2.2 Extraction of all PPIs/interrelationships and Reconstruction of Egr1-centered networks

In the next step of our strategy we combined the three previously discussed gene/protein lists and extracted all genetic and physical interactions and then we text-mined all published interrelationships from public databases with statistical tools that are incorporated within the websites, using an expectation value (E value) greater than 0.7. We verified that data reflected real interrelationships and rejected data that were mere co-incidences of textual referral. Thus, interaction data with an E value less than 0.7 were rejected (see Additional file 5).

### 2.3 Re-construction of 1× and 6× IMO stress networks

**Figure 2:**
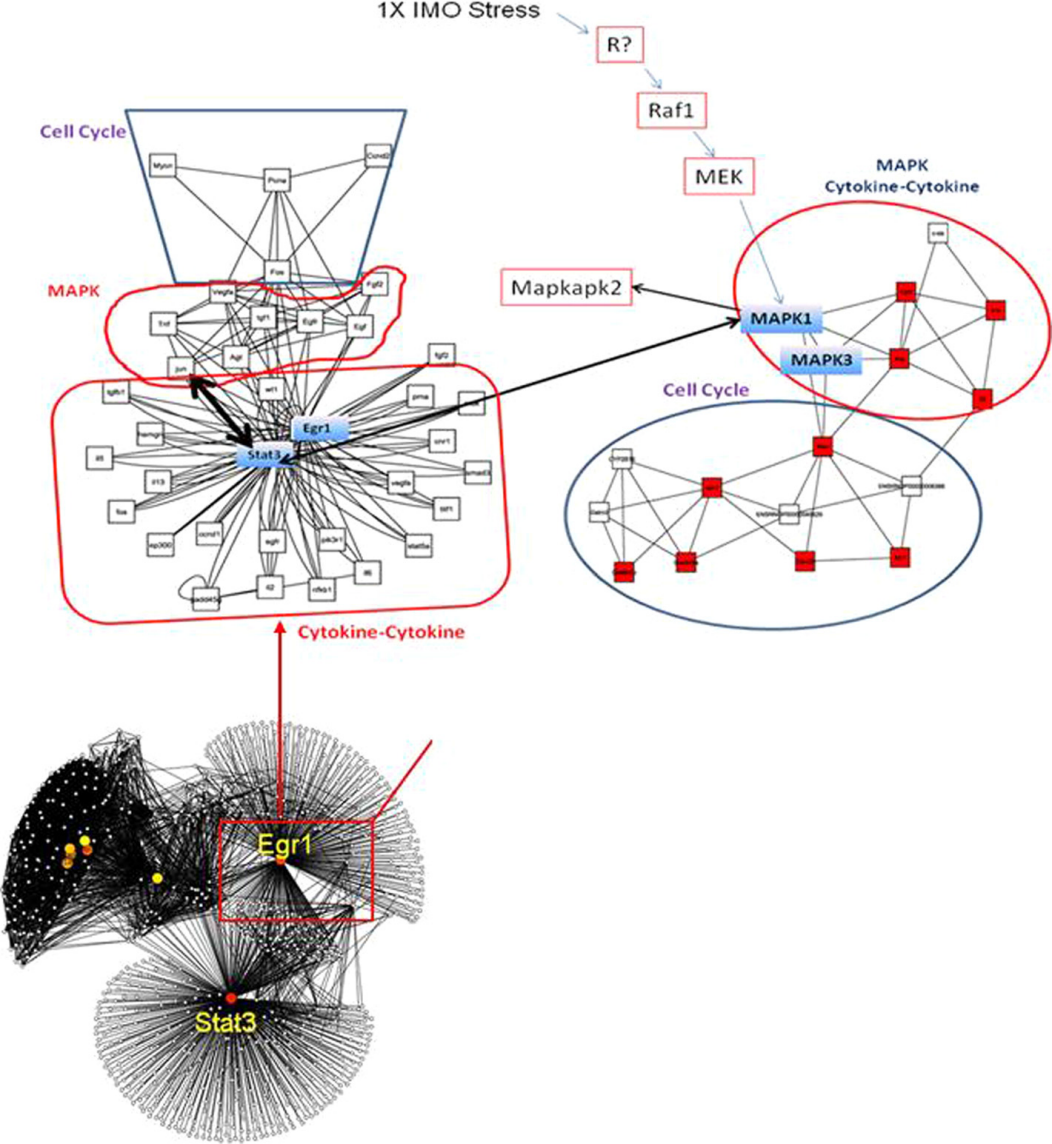
**Visual Representation of Acute (1×) Stress Network.** Upper panels: The 1× IMO network is reorganized around *Egr1* and *Stat3* and their links are grouped in terms of the top enriched functional modules cell cycle, cytokine-cytokine and MAPK. Network edges (black lines) link nodes (proteins/genes represented by open circles). Closest Egr1 network neighbors were calculated within the HUBBA website using Djikstra’s algorithm and were re-displayed with Cytoscape. Lower panel: The rat adrenal medulla 1**×** IMO full network contains 1717 genes/proteins (nodes) and 6554 interactions (edges) and is organized around Egr1 and Stat3 which are major hubs in this network. Note: upward pointing arrows (Prlh, Chgb, and Atp5b) indicate activation and downward pointing arrows (Egf) indicate suppression of expression.

**Figure 3:**
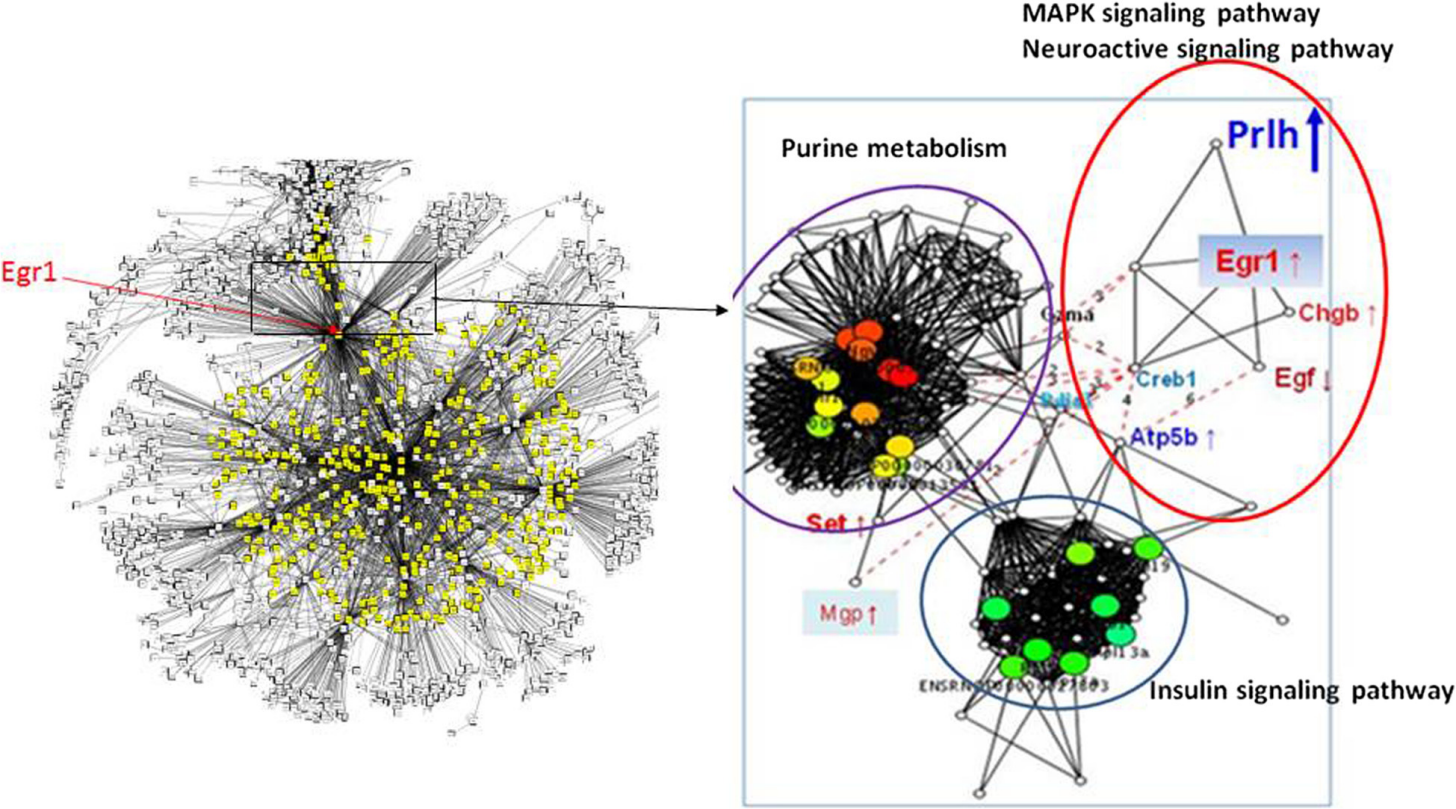
**Visual Representation of** r**epeated (6×) Stress Network.** Left panel: The rat adrenal medulla 6× IMO network contains 1313 genes/proteins (nodes) and 5203 interactions (edges). Genes/proteins that are first neighbors of *Egr1* were identified with Cytoscape and are in green. Right panel: Visual representation of the 6× IMO network edges (black lines) link nodes (proteins/genes represented by open squares). The *Egr1* neighborhood is shown in the right panel with first neighbors of *Egr1* in each motif (extracted with Cytoscape) shown in yellow and green. The nearest neighbors of *Egr1* and their shortest paths were calculated within the HUBBA website with Djikstra’s algorithm and re-displayed with Cytoscape. The arrows next to gene names indicate changed expression in 6× samples.

In the final step of our strategy we re-constructed 1× and 6× networks as described (Additional file 2: Computational and Bioinformatic Methods). The 1× network contains 1717 genes/proteins (nodes) and 6554 interactions (edges) and is whereas the 6× contains 1313 nodes and 5203 edges. It can be seen that the network parameters of 1× and 6× networks are similar to the parameters of other biological networks such as the yeast proteome or the human HTFN however, they are quite dissimilar to random ER networks (Table 1). We then re-displayed networks 1× and 6× around Egr1 and extracted the Egr1 network neighborhoods (Figure 2, left upper and right panels respectively, and Figure 3, left panel) within the HUBBA website by calculating the intersection between Egr1 and top hub and bottleneck nodes (see Additional file 6, “top hubs” Excel sheet). Notably, the 1× network is organized around Egr1, a result consistent with the central role of this transcription factor in acute stress, and also around Stat3, which is a novel observation (Figure 2, lower panel). In contrast, while Egr1 remains, as expected, a top hub in the 6× network, the other top hubs in this network, Apoa1 and Hspd1 are not neighbors of Egr1, suggesting that the Egr1 neighborhood is organized differently (Figure 3, right panel). In order to further analyze Egr1’s network neighbors in 1× and 6× networks, and infer possible links between them and Egr1 we extracted the top 10 motifs of interacting proteins with the MCODE algorithm within Cytoscape and identified top GO functional classifications and KEGG pathways with the GATHER algorithm for nodes in the neighborhood of Egr1. The Egr1-centered, 1× IMO network neighborhood motifs are enriched for cell cycle, MAPK kinase and cytokine pathways, consistent with their role in acute stress (Additional file 6 and Table 2), whereas the 6× Egr1-centered network motifs is enriched for purine metabolism, insulin signaling, MAPK and the neuroactive signaling pathways (Additional file 7 and Table 3).

**Table 1:** Topological properties of 1× and 6× IMO networks: Comparison with a random Erdos-Renyi network and with other biological networks

**Category**	**1×**	**6×**	**HTFN**^**1**^	**Yeast proteome**	**ER**^**2**^
N	1717	1313	230	1870	230
L	6554	5203	851	4488	851
(k)	3.8	3.9	3.7	2.4	3.7
(C)	0.15	0.1	0.17	0.07	0.015
l	2.407	2.984	4.5	6.81	4.15

N: Total number of nodes.

L: Total number of links (edges).

k: Average degree.

C: Average clustering.

l: Average path length.

^1^Human Transcription Factor Network.

^2^Erdos-Renyi.

**Table 2:** KEGG and GO pathways of top 10 Cytoscape modules in 1 × IMO network

	**Sub-network motif 1**	**Sub-network motif 2**	**Sub-network motif 3**	**Sub-network motif 4**	**Sub-network motif 5**
**KEGG pathway**	Cell cycle	Cell cycle	Cell cycle	Cytokine-cytokine receptor interaction	Complement and coagulation cascades
	MAPK signaling pathway	Apoptosis	TGF-beta signaling pathway	Cytokine-cytokine receptor interaction	Pentose and glucuronate interconversions
	Focal adhesion	MAPK signaling pathway	MAPK signaling pathway	Jak-STAT signaling pathway	Galactose metabolism
	Apoptosis	Focal adhesion	Wnt signaling pathway	MAPK signaling pathway	Citrate cycle (TCA cycle)
**GO pathway**	Cell cycle	Cell proliferation	Regulation of cell cycle	Response to biotic stimulus	Glycolysis
	Cell proliferation	Cell cycle	Cell cycle	Immune response	Exose catabolism
	Modification-dependent protein catabolism	Regulation of cell cycle	Cell proliferation	Defense response	Alcohol catabolism
	Ubiquitin-dependent protein catabolism	DNA-dependent DNA replication	Transcription, DNA-dependent	Organismal physiological process	Monosaccharide catabolism
	**Sub-network motif 6**	**Sub-network motif 7**	**Sub-network motif 8**	**Sub-network motif 9**	**Sub-network motif 10**
**KEGG pathway**	Cytokine-cytokine receptor interaction	Cytokine-cytokine receptor interaction	Cytokine-cytokine receptor interaction	MAPK signaling pathway	MAPK signaling pathway
	Cytokine-cytokine receptor interaction	ECM-receptor interaction	MAPK signaling pathway	MAPK signaling pathway	MAPK signaling pathway
	MAPK signaling pathway	Porphyrin and chlorophyll metabolism	Jak-STAT signaling pathway	MAPK signaling pathway	Toll-like receptor signaling pathway
	Jak-STAT signaling pathway		MAPK signaling pathway	Apoptosis	MAPK signaling pathway
**GO pathway**	Response to biotic stimulus	Transition metal ion transport	Response to biotic stimulus	Response to biotic stimulus	Protien amino acid phosphorylation
	Immune response	Di-, tri-valent inorganic cation transport	Immune response	Immune response	Morphogenesis
	Defense response	Metal ion transport	Defense response	Defense response	Phosphorylation
	Response to stress		Organismal physiological process	Response to stress	Protein kinase cascade

**Table 3:** **KEGG and GO pathways of top 5 Cytoscape modules in** 6× **IMO network**

	**Sub-network motif 1**	**Sub-network motif 2**	**Sub-network motif 3**	**Sub-network motif 4**	**Sub-network motif 5**
**KEGG pathway**	Insulin signaling pathway	Purine metabolism	Purine metabolism	Focal adhesion	Pyrimidine metabolism
	Focal adhesion	Purine metabolism	Purine metabolism	MAPK signaling pathway	Purine metabolism
	Regulation of actin cytoskeleton	Purine metabolism	Purine metabolism	MAPK signaling pathway	Pyrimidine metabolism
	TGF-beta signaling pathway	Pyrimidine metabolism	Pyrimidine metabolism	Jak-STAT signaling pathway	Purine metabolism
**GO pathway**	Protein biosynthesis	Cyclic nucleotide biosynthesis	cGMP metabolism	Morphogenesis	Nucleoside diphosphate metabolism
	Macromolecule biosynthesis	Cyclic nucleotide metabolism	cGMP biosynthesis	Development	Pyrimidine base metabolism
	Cellular biosynthesis	Nucleotide biosynthesis	Transcription initiation	Organogenesis	Deoxyribonucleoside diphosphate metabolism
	Biosynthesis	Nucleotide metabolism	Nucleobase, nucleoside, nucleotide	Organ development	Nucleotide metabolism

We further narrowed our focus on the shortest path neighbors of Egr1 within the top motifs in both acute and repeated stress. Egr1 and Stat3 also are top bottlenecks as well as network neighbors in the 1× network and they are in motif 4 (Additional file 6). Calculation of shortest paths in the neighborhood of Egr1within the HUBBA website revealed that Stat3 is a network neighbor of Egr1 (Figure 2, left upper panel) and Additional file 6, see “*top hubs*” sheet). Also, Egr1 is a top hub in 6× IMO samples, and is a member of motif 4 along with Prlh and Chgb (Figure 3, right panel). Few of the other nodes in other motifs were of sufficient interest for further functional analysis. We therefore extracted the intersection between top hubs and/bottlenecks and motif 4 members (Additional file 7) within HUBBA by calculating the shortest path between top hubs/bottlenecks and members of motif 4 which also contained Egr1 as well (Additional file 7, see sheet “*intersection*”). Notably, among Egr1’s neighbors, expression of Prlh and Chgb were also up-regulated but not that of CREB (Additional file 7, “intersection” Excel sheet).

Gene ontology analysis of 1× network motifs and the Egr1/Stat3 neighborhood showed that it is enriched for cell cycle, cell proliferation and cytokine-cytokine receptor (Sub-network motifs 4, 6, 7, 8) and Jak/Stat pathway genes (Additional file 6) whereas 6× network motifs are enriched for genes belonging to the insulin signaling, neuroactive signaling and the purine metabolism pathways. Prlh11 is a network neighbor of Egr1 (Figure 3, right panel). Strikingly, the MAPK signaling pathway is enriched for genes in all motifs except 5 and 7 (1× network) an observation that is consistent with previous results. This finding is consistent with a role of the ERK-STAT3-Egr1 pathway in neurite outgrowth. Specifically, MEK-ERK1/2 is required for FGF1-induced neurite outgrowth, pSTAT3 (S727) and Egr1 expression in PC12 cells [27].

**Figure 4:**
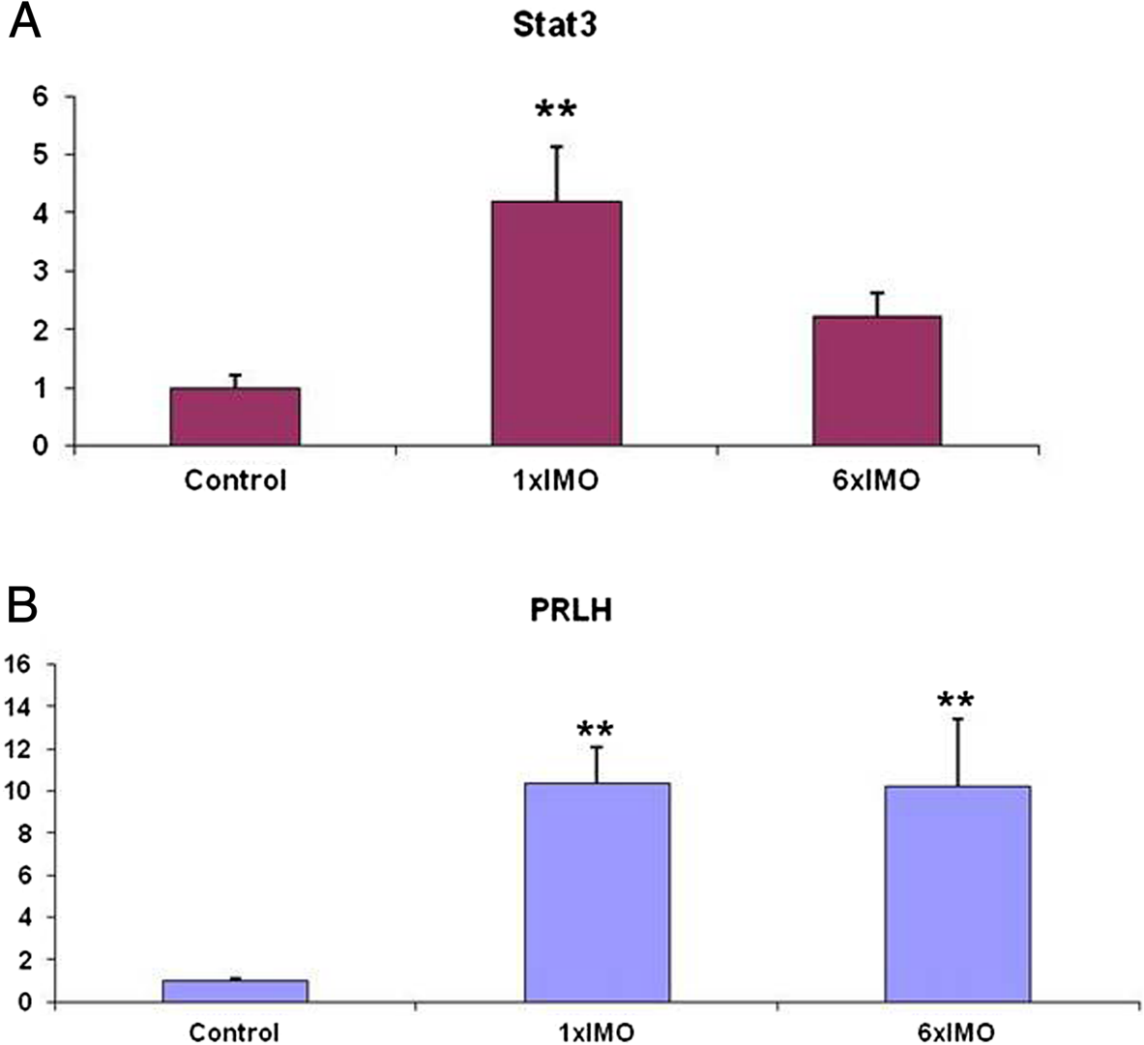
**mRNA levels of Stat3 (panel A) and Prlh1 (panel B) in control, 1× or 6× IMO stress samples, were detected with real time PCR.** Fold induction is on the x axis and category on the y axis. Statistical significance was determined as described in Methods. **p ≤ 0.01 compared to control.

In order to confirm our findings, we tested independent samples of 1× and 6× *Egr1* network neighbors for expression changes with qRT-PCR or immunoblots (Tables 4 and 5). Several of these changes were further verified in independent IMO stress samples at the mRNA level by qRT-PCR or at the protein level by western blot analysis or immunocytochemistry (Table 4). These findings confirm for the first time that *Stat 3* expression (Figure 4, panel A) is significantly up-regulated with 1× IMO. Expression of Prlh1 (and of Chgb) is up-regulated in both 1× and in 6× (Figure 4, panel B). Strikingly, we observed for the first time down regulation of gene PIAS3 (*Inhibitor of Stat 3*, Table 4, bottom) implicating Stat3 signaling in acute IMO stress activation. PIAS3 is a small E3-type small ubiquitin-like modifier (SUMO) ligase that plays a critical role in regulating the Stat3 signaling pathway by inhibiting Stat3-DNA binding [28],[29]. The *Egr1* neighbors *Prlh11* (and *Chgb*) are significantly represented in changes in the 6× IMO samples, suggesting that they might have important functions in long-term stress signaling (Figure 4, panel B).

**Table 4:** Changes in Gene expression of key factors in 1× IMO samples

**Name**	**Change via microarray**	**mRNA verified**	**Protein verified**
Egr1	⬆⬆⬆	⬆⬆⬆	⬆ [25],[30]
CREM	⬆⬆⬆	-	-
DBH	Not changed	⬆ [2]	Not changed
DUSP14	⬆⬆	-	-
Egfr	⬆⬆	⬆⬆	-
Fos	⬆⬆⬆	⬆⬆⬆	Yes [31],[32]
Jun	⬆⬆	⬆	-
MAPKAP2	⬆⬆	⬆⬆⬆	-
Stat3	⬆⬆	⬆⬆	-
Inhibitor of activated Stat3 (PIAS3)	⬆	-	-

Upward or downward-pointing arrows symbolize the following: ⬆changed up to 2.5 fold. ⬆⬆ 2.5 to 10.0 fold. ⬆⬆⬆ greater than 10.0 fold. Changes in mRNA levels were confirmed by Northern Blot, qRT-PCR or Real Time PCR array.

**Table 5:** Changes in gene expression of key factors in 6× IMO samples

**Name**	**Change via microarray**	**mRNA verified**	**Protein verified**
Egr1	⬆⬆	⬆⬆	⬆ [25],[30]
CREB	Not changed	Not changed	Increased Phosphorylation ⬆⬆ [33]
Chromogranin B	Not changed	⬆	-
DBH	Not changed	⬆⬆ [2]	⬆⬆
Egfr	Not changed	Not changed	-
PNMT	Not changed	⬆⬆ [3],[4]	⬆ [34]
MAPKAP2	⬆⬆	⬆⬆	-
Prlh	⬆⬆	⬆⬆⬆	-

Upward-pointing arrows symbolize the following: ⬆changed up to 2.5 fold. ⬆⬆ 2.5 to 10.0 fold. ⬆⬆⬆ greater than 10.0 fold. Changes in mRNA levels were confirmed by Northern Blot, qRT-PCR or Real Time PCR array.

## 3 Discussion

We have sought to identify novel members in pathways, particularly transcription factor pathways, and gene products that might be regulated in IMO stress. We approached this by performing microarray analyses of samples derived from the adrenal medullae of rats subjected to acute (1×) or repeated (6×) IMO stress in order to identify genes/gene products that are co-expressed or co-regulated with the transcription factor Egr1, which is critical for stress responses in the adrenal medulla of rats. Following identification of gene modules that are co-expressed with Egr1 with KS ranking and GSEA analysis, we mined all physiologically relevant interactions and interrelationships with text-mining, and we established that Stat3 and Prlh are significantly up-regulated in 1× and in 6× IMO stress responses suggesting for the first time that they are likely participants. Also, we confirmed earlier observations that Chgb is activated in 6× IMO stress [35]. Stat3 is co-expressed and a network neighbor of Egr1 in the re-constructed 1× network, implicating Stat3 involvement in IMO stress. Notably, the 1× network is organized around Egr1 and Stat3 since they are top hubs (highest number of interactions/interrelationships, Figure 2, lower panel). This contention is supported by the concomitant down-regulation of the Stat3 inhibitor PIAS3 which inhibits the DNA activity of Stat3 therefore shutting down part of Stat3-mediated genomic expression changes. The observed enrichment for MAPK and cytokine genes in 1× IMO stress (see Figure 2) is consistent with the involvement of cytokines in regulating the stress response. Stat3 belongs to the STAT (signal transducers and activators of transcription) family of transcription factors that feed into the Jak/Stat signaling cascade. Several cytokine receptors regulate this cascade and this is consistent with the observed GO enrichment for cytokine receptors. Stats are phosphorylated by Jaks and activated, allowing them to translocate to the nucleus. The Jak/Stat pathway is regulated by phosphorylation/dephosphorylation by kinase/phosphatase enzymes, by *Stat* gene activation antagonists such as SOCS (suppressors of cytokine signaling) and by PIAS (Protein Inhibitors of Activated Stats) [36],[37].

Prlh1 is a peptide widely distributed in the CNS and involved in mediating stress responses and activating the HPA axis [38]. Prlh1 was found to be co-expressed with TH and PNMT in Epi synthesizing cells of the adrenal medulla [39], however its function in the adrenal is unclear.

In repeated stress (6× IMO), the Egr1-centered network is organized differently with Stat3 being absent and with Prlh and Chgb being close network neighbors of Egr1 (Figure 3, right panel). Moreover, the enriched motifs are strikingly different compared to the 1× Egr1 neighborhoods. The predominant motifs include members of the insulin signaling pathway and members of pathways for purine metabolism, neuroactive signaling and MAPK pathway. The latter observation is in agreement with the central role of the MAPK pathway in both acute and repeated stress responses. Chgb is up-regulated confirming previous experiments (see ref [35]). Induction of chromogranin B (Chgb) gene expression selectively with 6× IMO stress but not with 1×, is especially intriguing since Chgb functions in the biogenesis of secretory granules and in the sorting of proteins to the regulated secretory pathway [40]. Chgb deficient mice display reduced levels of catecholamines released per quanta [41]. The induction of Chgb with repeated IMO suggests formation of additional neurosecretory vesicles or production of larger quantal release which may help provide additional neurosecretory strength to adapt to further demands of chronically repeated stress [35]. The induction of Chgb with repeated IMO suggests that formation of additional neurosecretory vesicles or production of larger quantal release may help provide additional neurosecretory strength to adapt to further demands of chronically repeated stress.

## 4 Conclusions

In addition to the transcription factor Egr1 which is critical for IMO stress induction in the rat adrenal medulla, we here provide evidence for the first time that the gene encoding the transcription factor Stat3 and the gene encoding the peptide Prlh1 are activated in acute (1×) and in repeated stress (6×) respectively. The data suggest that the transcription factor Egr1 has different roles in acute and in repeated stress, indicated by different networks and network neighbors and furthermore that Stat3 signaling in 1× and Prlh (and Chgb) activation cascades in 6× might also be important in transitioning from acute to repeated stress responses.

## 5 Methods

### 5.1 Animal methods

The stress procedures, isolation of RNA and Afflymetrix analysis were as previously described [6]. Briefly, male, murine pathogen-free, Sprague–Dawley rats (280–320 g), obtained from Taconic Farms (Germantown, NY, USA), were maintained under controlled conditions of a 12 h light–dark cycle (lights on from 6 am to 6 pm) at 23 ± 2°C with food and water *ad libitum.* All animal experiments were performed in accordance with the NIH Guide for the Care and Use of Laboratory Animals and approved by the Institutional Animal Care and Use Committee.

Immobilization stress (IMO) was performed as previously described [2],[26],[42]. For acute stress rats were subjected to immobilization stress for 2 hrs once (1 × IMO). For repeated stress, the animals were immobilized for 2 hrs daily for 6 consecutive days (6 × IMO). Following the last IMO, rats were euthanized by decapitation the adrenal medulla dissected. Control groups were not exposed to stress (absolute controls). All animal manipulations were performed between 8 AM and 1 PM to control for circadian variations.

### 5.2 RNA isolation

For the microarray, RNA was isolated from two separate immobilization experiments. To minimize sample variability caused by individual differences among animals, each sample was pooled from left adrenal medulla from 4 individual rats. There were 3 pooled samples for each group. RNA was extracted using Absolutely RNA Miniprep Kit (Stratagene, La Jolla, CA). The integrity of the RNA was assessed by the A260/A280 ratio which was close to 2.0 and by electrophoresis (Agilent Bioanalyzer 2100).

### 5.3 Microarray analyses

Gene expression analyses were performed by the NIH Neuroscience Microarray Consortium at UCLA Medical Center (Los Angeles, CA). Total RNA (≥4 μg) from each group was converted to cDNA by using superscript reverse transcriptase and the T7-Oligo (dT) promoter primer kit (Affymetrix, Inc). Following RNase H-mediated second-strand cDNA synthesis, the double-stranded cDNA were purified and served as a template in the subsequent *in vitro* transcription reaction (Affymetrix, Inc.). The *in vitro* transcription reaction was carried by T7 RNA polymerase and a biotinylated nucleotide analog/ribonucleotide mix (Affymetrix, Inc.). The biotinylated cRNA targets were purified and fragmented. Each cRNA was hybridized to an individual Affymetrix GeneChip Rat Array Expression 230 2.0 (RAE 230 2.0 array) which was subsequently processed for washing and staining with the antibody stain solution with streptavidin phycoerythrin and the arrays were scanned on the GeneChip Scanner 3000. The raw pixel data have been deposited to the Gene Expression Omnibus (GEO) database Series # GSE8184.

### 5.4 Computational and bioinformatic methods

Top genes in gene sets that might be co-regulated with Egr1 were identified with Kolmogorov-Smirnov (KS) statistics and gene set enrichment analysis (GSEA) [43],[44] (For full description, see Additional file 2) on the genome-wide gene expression microarray data sets obtained from samples of the adrenal medulla of rats subjected to 1× or 6× IMO stress, each data set containing three samples. The strategy employed extraction of genes that are co-expressed with Egr1 in single (1×) or repeated (6×) exposure to stress (Figure 1).

### 5.5 Statistical methods

Animal experiments with n = 4 per group were from at least two separate experiments. Data were analyzed with ANOVA followed by Bonferroni post-hoc analysis using GraphPad Prism 4 software (GraphPad Software, Inc., La Jolla, CA). A value of p = ≤ 0.05 was considered significant.

## Abbreviations

ANOVA: Analysis of variance

DBH: Dopamine β-hydroxylase

Egf: Epidermal growth factor

EgfR: Epidermal growth factor receptor

Egr1: Early growth factor 1

Egr1_POS: *Egr1* positive gene index phenotype

Epi: Epinephrine

ER: Erdos-Renyi

ES: Enrichment score

Fgf2: Fibroblast growth factor 2

GATHER: Gene annotation tool to help explain relationships

GSEA: Gene set enrichment analysis

Gzma: Granzyme A

*HTFN*: Human transcription factor network

HUBBA: Hub objects analyzer

*Il5*: Interleukin *5*

*Il6*: Interleukin *6*

IMO: Immobilization stress

Jak: Janus Kinase

1 × IMO: Single immobilization stress/acute IMO stress

6 × IMO: Immobilization stress repeated daily for 6 consecutive days/prolonged IMO stress

KS: Kolmogorov-Smirnov

MAP: Mitogen-activated protein

Mgp: Matrix Gia Protein

NE: Norepinephrine

SOCS: Suppressors of cytokine signaling

PNMT: Phenylethanolamine N-methyltransferase

Prlh1: Prolactin releasing peptide

PIAS3: Protein inhibitor of activated Stat3

RT-PCR: Real time polymerase Chain reaction

Stat3: Signal transducer and activator of transcription 3

TH: Tyrosine hydroxylase

## Competing interest

The authors declare that they have no competing interests.

## Authors’ contributions

NAP conceived the idea, executed all computational and bioinformatic methods (KS statistics, module analysis with GSEA, GO analysis and network re-construction) and oversaw all aspects of the work and the manuscript. AT and XL performed all experiments except for microarray experiments which were performed at the NIH Neuroscience Microarray Consortium, UCLA Medical Center, CA. AGP has assisted with interpretation of the results and with the manuscript and ELS has managed all aspects of the work. All authors read and approved the final manuscript.

## Additional files

## Supplementary Material

Additional file 1: Figure S1. Schematic representation of the KS ranking and GSEA procedures. The normalized enrichment score (ES) can be positive (for gene sets that are enriched and therefore correlate with the expression profile) or negative for anti-correlating profiles. Click here for file

Additional file 2: Computational and bioinformatic methods, specifically for Kolomogorov-Smirniv statistics and for gene set expression analysis. Click here for file

Additional file 3: **GSEA 1× IMO data.** This file contains the GSEA parameters used in analyzing 1× microarray samples, the ranked gene list and their KS scores, the top Egr1 gene index and categorical gene lists and their corresponding top gene sets with their ES scores. Click here for file

Additional file 4: **GSEA 6× IMO data.** Same as for Additional file 3, except the data are for 6× samples. Click here for file

Additional file 5: **Gene Interaction-Interrelationship Modules.** This file contains the two gene module lists generated with GSEA for 1× and 6× samples respectively, and the list of Egr1 co-expressed genes extracted from the CoxpressDb. Click here for file

Additional file 6: **1× IMO Network Motif GO Categories and Network Data.** Additional file 6 contains the top motifs for 1× IMO samples, the Gene Ontology enrichment data for the motifs, motif figures, top hubs and bottlenecks. Click here for file

Additional file 7: **6× IMO Network Motif GO Categories and Network Data.** Same as Additional file 5. Click here for file
