# Supplementary figures and images for: A systems approach identifies co-signaling molecules of early growth response 1 transcription factor in immobilization stress
Source: BMC Syst Biol. 2014 Sep 11;8:100. doi: 10.1186/s12918-014-0100-8 (PMC4363937; doi:10.1186/s12918-014-0100-8)

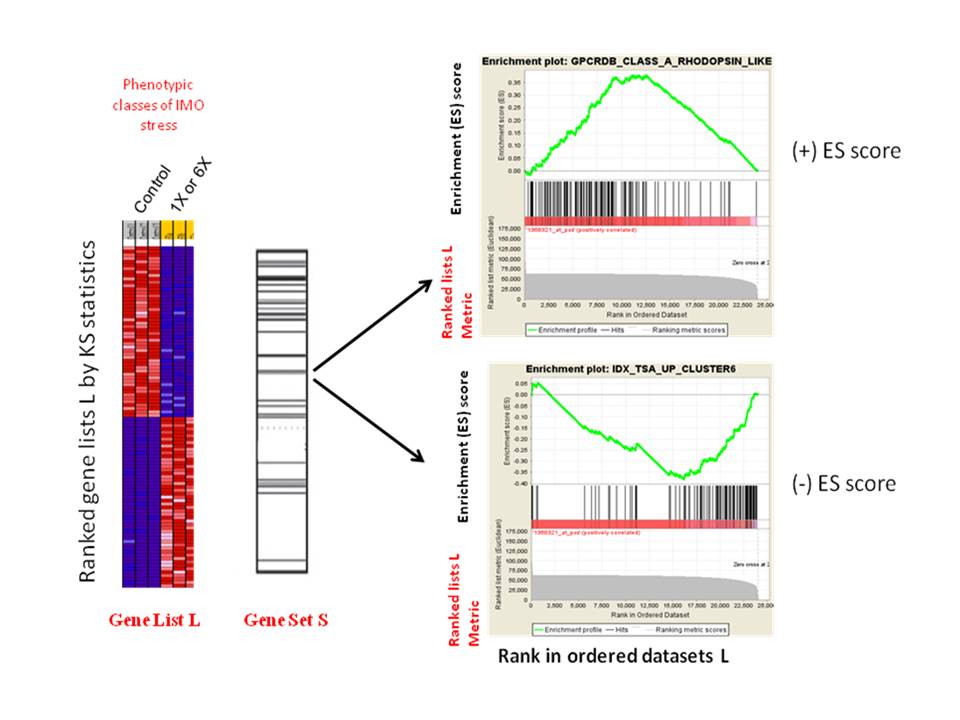

Supplement: Additional file 1: Figure S1. — Schematic representation of the KS ranking and GSEA procedures. The normalized enrichment score (ES) can be positive (for gene sets that are enriched and therefore correlate with the expression profile) or negative for anti-correlating profiles. [file s12918-014-0100-8-S1.jpeg]
