# Supplementary material for: A systems approach identifies co-signaling molecules of early growth response 1 transcription factor in immobilization stress
Source: BMC Syst Biol. 2014 Sep 11;8:100. doi: 10.1186/s12918-014-0100-8 (PMC4363937; doi:10.1186/s12918-014-0100-8)
Supplement: Additional file 2: — Computational and bioinformatic methods, specifically for Kolomogorov-Smirniv statistics and for gene set expression analysis. [file s12918-014-0100-8-S2.docx]

# Additional Files

**A Systems Approach Identifies Co-Signaling Molecules of Early Growth Response 1 Transcription Factor in Immobilization Stress**

Nikolaos A. Papanikolaou^1,2, *^, Andrej Tillinger^3^,Xiaoping Liu^3,**^, Athanasios G. Papavassiliou^4^ and Esther L. Sabban^5^

Contents

[Supporting Information 1](#_Toc348434081)

[Kolmogorov-Smirnov (KS) ranking of gene expression and GSEA identification of Egr1 co-expressed gene modules 2](#_Toc348434082)

[GSEA Parameters 3](#_Toc348434083)

[Extraction of all PPIs/ interrelationships and Reconstruction of Egr1-centered networks 4](#_Toc348434084)

[Reconstruction of 1x and 6x IMO stress Egr1 networks from PPI and Gene Interrelationships 4](#_Toc348434085)

[Extraction of Network Motifs 7](#_Toc348434086)

[Gene ontology (GO) enrichment analysis 7](#_Toc348434087)

[References 8](#_Toc348434088)

## Kolmogorov-Smirnov (KS) ranking of gene expression and GSEA identification of Egr1 co-expressed gene modules

In order to extract gene modules that are co-expressed with Egr1 in acute (1x) or prolonged (6x) IMO stress samples, we first ranked all genes in the 1x and 6x IMO datasets with respect to expression levels using KS statistics, a non-parametric approach that makes no assumptions about the distribution of expression values between or within classes, with the KS module using Gene Pattern (<http://www.broadinstitute.org/cancer/software/genepattern/>) ([Reich, et al., 2006](#_ENREF_5)). For N genes in each class *c(k) and t(k)* represent the *k*^th^ gene from the control and test populations respectively where k=1,…..K, ∑ ^K/^ _k=1_ *c(k)* =1, and ∑*t(k)* =1. The cumulative distribution function (CDF) for the control and test classes are defined as *C(j)* and *T(j)* and computed with C(*j*)= ∑k=*_1 -J_ c(k)*, T(*j*) =*_1- J_ t(k)*. Finally, the KS statistic, which is the maximal distance between the two CDFs is computed with D=max *j* │C(*j*)-T(*j*)│producing gene lists L. The KS statistic is a quantitative measure of how likely the observed expression distribution differences between control and 1x or control and 6x datasets is due to random chance.

Next we analyzed 1x and 6x microarray data with gene set enrichment (GSEA) analysis using two options: First, using Egr1 as a gene index with a Euclidean distance metric and calculated the median of probe values, controls vs. 1x or controls vs. 6x, Additional Files1 and 2 for details) we extracted the top 100 genes whose expression profile is statistically similar to that of Egr1 and second, using categorical analysis to extract the top fifty up- or down-regulated genes essentially as described ([www.broad.mit.edu/gsea/index.jsp](http://www.broad.mit.edu/gsea/index.jsp)) (See Additional Files1 and 2 for details on statistics). GSEA calculates an enrichment score, ES ((supplementary figure 1)), for the position of any gene in the KS-ranked lists L. The ES score reflects the position of each gene within the ranked lists L and it was computed in accordance to the non-parametric rank statistic X, where X is the number of genes in the query gene sets from control, 1x or 6x IMO stress classes, Z is the number of genes (31099 genes/proteins) in the ordered list and Y=Z-X (see Additional Files1 and 2 for detailed GSEA conditions and for gene sets used as well as for a summary of the retrieved gene sets). The ES score for *Egr1* is algorithmically set to zero by default (when used as an index gene for extracting top co-expression neighbors) and the rank position of each gene in the ordered 1x or 6x array was computed. The closest scores designate genes within the enriched gene sets that are closest in profile to *Egr1* and the largest scores the most distant genes. The algorithm then calculates a vector V, where V(i) is the statistic corresponding to any gene in the ordered list having value V(i)=+Y, if gene i is in the gene set and V(i)=-X if it is not. Also, false discovery rate (FDR) and p value statistics are computed for each gene in the ranked lists L of the two classes. Gene sets with significantly positive ES scores are those where V(i)=+Y whereas those with negative ES are V(i)=-X. KS-ranked genes, GSEA-derived heat maps of top categorical/Egr1-gene index-derived genes and gene sets are shown in Additional Files1 (1x IMO stress) and 2 (6x IMO stress).

## GSEA Parameters

GSEA was executed on the ranked gene lists L in terms of the two class vectors, control vs. 1x or control vs. 6x IMO stress using *Egr1* as an index gene (i.e. its expression level of its probe set across all six samples in the two classes), and Euclidean distance as the gene distance metric (Expression datasets I with N genes and k samples, where k=6 for 1x or 6x IMO samples), and computed enrichment scores (ES) for each gene in S relative to its position in the ordered gene lists L (Figure S1 and Additional Files1 and 2). We generated a total of six gene co-expression modules: Four with GSEA: Two lists of top 100 genes co-expressed with Egr1, Egr1 as an index gene for 1x and 6x respectively and two categorical lists for 1x and 6x respectively, and one containing three hundred genes reported in the literature to be co-expressed with egr1, from the co-expression database COXPRESdb database (<http://coxpresdb.jp/>) (Additional File 3). More specifically, using categorical analysis, the top fifty down-regulated and top fifty up-regulated co-expressed genes were computed with GSEA for 1x and 6x samples. The GSEA algorithm computes whether genes in S are ranked high (positive ES) or low (negative ES) in L, and returns a numeric value representing the positional distribution of the entire set of query genes in the two classes (Figure S1).

## Extraction of all PPIs and Functional interrelationships: Reconstruction of Egr1-centered networks

We combined the four gene module lists (shown in Additional File3) and used them to extract all genetic/physical interactions and text-mined all published interrelationships from public databases using an expectation value (E value) greater than 0.7. We verified that data reflected real interrelationships and rejected data that were mere co-incidences of textual referral. Also, interaction data with an E value less than 0.7 were rejected.

## Reconstruction of 1x and 6x IMO stress Egr1 networks from PPI and Gene Interrelationships

Protein-protein interactions between the proteins encoded by genes in the three gene lists (gene modules) were generated with GSEA and one from the Coxpresdb database were extracted from the HPRD, STRING, DIP and i2d databases. Interrelatioships (genetic interactions) among the genes in the three groups of gene modules (gene lists) that were generated with GSEA and from the cocpresdb, were extracted from the CONRAD database ([www.conrad.licr.org](http://www.conrad.licr.org)). The interaction and interrelation data were combined and analyzed for functional module and complex retrieval with the Cytoscape Program ([www.cytoscape.org/](http://www.cytoscape.org/) ([Shannon, et al., 2003](#_ENREF_6)), an open source bioinformatics software program that allows network reconstruction as well as identification of network motifs and multiprotein complex modules with the embedded MCODE algorithm. First, 1x or 6x, *Egr1*-centered, undirected networks were re-constructed and the direct interactors of *Egr1* extracted. Also, the top 10 protein complexes (motifs) for 1x IMO and 5 for 6x samples were identified and extracted using the MCODE plugin ([Bader and Hogue, 2003](#_ENREF_1)).

In order to re-construct 1x and 6x Egr1 networks, genes in the *Egr1* gene index and categorical (control vs. IMO stress samples) EGR1_POS modules that contain *Egr1* expression neighbors as well as genes extracted from the COXPRESdb database, were used as queries to mine all their interactions from protein-protein interaction databases STRING (http://string.embl.de/), HPRD (<http://www.hprd.org/index_html>), DIP (http://dip.doe-mbi.ucla.edu/dip/Main.cgi) and i2d (http://ophid.utoronto.ca/ophidv2.201/). Also all high confidence (c>0.7) interrelationships in the pSTIING database were mined (<http://pstiing.licr.org/>, ([Ng, et al., 2006](#_ENREF_4)). Next, networks for 1x or 6x were re-constructed from these data and displayed with the Cytoscape program (http://www.cytoscape.org/), or within the VisANT (http://visant.bu.edu/) and Hub Objects Analyzer (Hubba, (<http://hub.iis.sinica.edu.tw/Hubba/>) websites. Top hubs, bottlenecks and network neighbors of *Egr1* were calculated with the Djiskstra algorithm in Hubba or Cytoscape as described in the next paragraph and top candidates were identified (Additional Files4 and 5).

The 1x network contains 1717 genes/proteins (nodes) and 6554 interactions (edges) whereas the 6x contains 1313 nodes and 5203 edges (in Table 1 we compare them to a random ER network and to the HTFN network). We then re-displayed the networks around Egr1 and extracted the Egr1 network neighborhoods (supporting information Figures S2, left upper panel and right panels respectively; supporting information fig S3, left panel) within the HUBBA website by calculating the intersection between Egr1 and top hub and bottleneck nodes (see supporting information for details on the algorithms used). In order to further analyze Egr1’s network neighbors and infer possible links between them and Egr1 we extracted the top 10 motifs of interacting proteins with the MCODE algorithm within Cytoscape (Additional Files4 and 5) and identified top GO functional classifications and KEGG pathways with the GATHER algorithm (Additional Files4 and 5 and Tables 2 and 4) for nodes in the neighborhood of Egr1.

We then extracted the top 1x or 6x hubs and the top bottlenecks using the k method within the HUBBA website. We identified top neighbors of *Egr1* by using the web window interface “Particular group” within HUBBA, which uses Dijkstra’s algorithm and returns the network neighbors of entered nodes and their links to additional, chosen nodes in the network (in this case *Egr1*) and calculated the shortest paths between top hubs and *Egr1* network neighborhood nodes. Also, we searched and retrieved links between top network hubs and members of the top 10 complexes (motifs), and in particular links with members of complexes which contain *Egr1*.

## Extraction of Network Motifs

We extracted the top motifs within Cytoscape using the MCODE algorithm which identifies known protein-protein interactions (Additional Files4 and 5). Next, within the HUBBA website we cross-extracted top bottlenecks and hubs that are neighbors of Egr1 and of motif members as decribed in the previous paragraph.

## Gene ontology (GO) enrichment analysis

Enriched gene ontology (GO) terms for *Egr1* neighbors in 1x or 6x networks, which were identified as described in the previous section, were extracted within Cytoscape using the BINGO algorithm ([Maere, et al., 2005](#_ENREF_3)) and confirmed with the GATHER web-site based algorithm ([Chang and Nevins, 2006](#_ENREF_2)). GATHER uses several different functional annotation tools to evaluate the statistical significance of enriched functional annotations of several categories such as KEGG pathways, MeSH, transcription factor binding sites etc, combined with evolutionary homolog and network-predicted annotations for proteins related through literature or protein interactions. The chief evaluation tool is the Bayes factor, a numerical indicator of significance with high positive numbers indicating high significance.

## References

Bader, G.D. and Hogue, C.W. (2003) An automated method for finding molecular complexes in large protein interaction networks, *BMC Bioinformatics*, **4**, 2.

Chang, J.T. and Nevins, J.R. (2006) GATHER: a systems approach to interpreting genomic signatures, *Bioinformatics (Oxford, England)*, **22**, 2926-2933.

Maere, S., Heymans, K. and Kuiper, M. (2005) BiNGO: a Cytoscape plugin to assess overrepresentation of gene ontology categories in biological networks, *Bioinformatics*, **21**, 3448-3449.

Ng, A.*, et al.* (2006) pSTIING: a 'systems' approach towards integrating signalling pathways, interaction and transcriptional regulatory networks in inflammation and cancer, *Nucleic Acids Res*, **34**, D527-534.

Reich, M.*, et al.* (2006) GenePattern 2.0, *Nat Genet*, **38**, 500-501.

Shannon, P.*, et al.* (2003) Cytoscape: a software environment for integrated models of biomolecular interaction networks, *Genome Res*, **13**, 2498-2504.
